# The Value of Pepsin

**Published:** 1874-10

**Authors:** 


*The Value of Pepsin.*—In the *Boston Medical and Surgical Journal,* of January 1st, Dr. R. T. Edes describes some careful experiments with the various kinds of pepsin, from which he arrives at the following conclusions:

Much of the dissatisfaction with pepsin expressed by physicians is due to the use of preparations which contain little or none of it.

The pepsin made by Scheffer's process, is by far superior to any other in ordinary use.

The wine is feeble, but not necessarily inert.

Elixirs of pepsin and bismuth are humbugs.

Pepsin should be administered with an acid, and with as few drugs as possible. A small amount of alcohol is not inadmissible, but a large amount retards digestion.

Its beneficial action is *not limited* by the amount of albumen which it dissolves in a test-tube without change or renewal of any of the contents.—*N. Y. Med. Jour.*

